# Neuroimmunology of Cardiovascular Disease

**DOI:** 10.1007/s11906-024-01301-8

**Published:** 2024-04-13

**Authors:** Sara M. Zarate, Annet Kirabo, Antentor O. Hinton Jr., Monica M. Santisteban

**Affiliations:** 1 Division of Clinical Pharmacology, Department of Medicine, Vanderbilt University Medical Center, Nashville, USA; 2 Department of Molecular Physiology and Biophysics, Vanderbilt University, Nashville, USA; 3 Vanderbilt Center for Immunobiology, Nashville, USA; 4 Vanderbilt Institute for Infection, Immunology, and Inflammation, Nashville, USA; 5 Vanderbilt Institute for Global Health, Nashville, USA; 6 Vanderbilt Memory and Alzheimer’s Center, Vanderbilt University Medical Center, Nashville, USA

**Keywords:** Neuroimmunology, Border associated immune cells, Neuroinflammation, Cardiovascular disease, Hypertension

## Abstract

**Purpose of Review:**

Cardiovascular disease (CVD) is a leading cause of death and chronic disability worldwide. Yet, despite extensive intervention strategies the number of persons affected by CVD continues to rise. Thus, there is great interest in unveiling novel mechanisms that may lead to new treatments. Considering this dilemma, recent focus has turned to the neuroimmune mechanisms involved in CVD pathology leading to a deeper understanding of the brain’s involvement in disease pathology. This review provides an overview of new and salient findings regarding the neuroimmune mechanisms that contribute to CVD.

**Recent Findings:**

The brain contains neuroimmune niches comprised of glia in the parenchyma and immune cells at the brain’s borders, and there is strong evidence that these neuroimmune niches are important in both health and disease. Mechanistic studies suggest that the activation of glia and immune cells in these niches modulates CVD progression in hypertension and heart failure and contributes to the inevitable end-organ damage to the brain.

**Summary:**

This review provides evidence supporting the role of neuroimmune niches in CVD progression. However, additional research is needed to understand the effects of prolonged neuroimmune activation on brain function.

## Introduction

The brain was once thought to be immune privileged, but it is now recognized to contain major immune niches along its borders that result from the infiltration of immune cells into the choroid plexus, meninges, and spinal cord. This occurs in the absence of inflammation, suggesting that in addition to a role in disease pathology [1], these immune niches are important for brain development [2] and homeostasis [3]. Over the last two decades, researchers have demonstrated long-range communication between the brain and immune system [4] via drainage of cerebrospinal fluid (CSF) and immune cells drain through meningeal lymphatic vessels into the deep cervical lymph nodes. More recent evidence has shown that the skull bone marrow serves an important role in central nervous system (CNS) immune surveillance in normal physiology and the response to injury [5]. Furthermore, a number of cytokines and pro-inflammatory signaling pathways are implicated in hypertension and other cardiovascular diseases (CVD) [6]. Thus, rather than being shielded from the immune system, the brain maintains a dynamic and functional relationship with the immune system for the homeostatic regulation of the brain and the body.

**Fig. 1:**
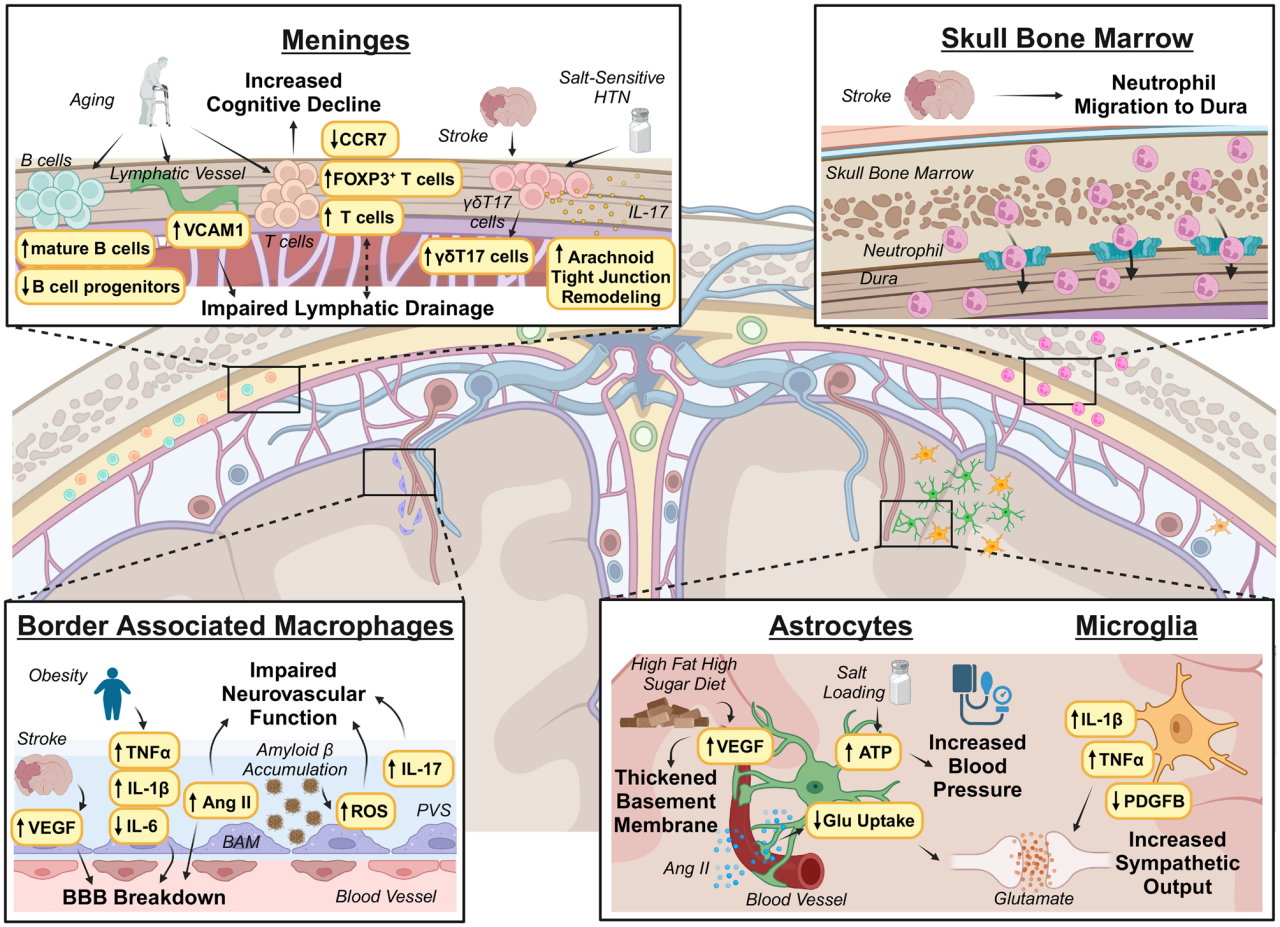
Neuroimmune niches respond to and contribute to the effects of cardiovascular disease (CVD) in the brain. Meninges: Aging is associated with B-cell expansion in the dura as well as changes to T-cell localization in the dura caused by decreasing expression of the homing receptor CCR7 and by increasing the density of FOXP3^+^ T-cells that reduce immune responses like waste clearing. Increased expression of VCAM1 in aging lymphatic vessels leads to impaired lymphatic drainage, which may also contribute to T cell accumulation in the aging dura. In salt-sensitive hypertension (HTN), γδT17 cells in the dura release the pro-inflammatory cytokine IL-17 which enters the cerebrospinal fluid through a disrupted arachnoid barrier. Skull bone marrow: After stroke, skull bone marrow-derived neutrophils migrate through direct vascular channels into the dura and later to the infarct site. Border associated macrophages: HTN, stroke, and obesity can contribute to blood–brain barrier breakdown by increasing expression of VEGF or pro-inflammatory cytokines such as TNFα and IL-1β. Accumulation of toxic protein aggregates like amyloid B can increase ROS causing impaired neurovascular function. Astrocytes and microglia: Astrocyte exposure to a high fat high sugar diet can thicken the basement membrane around cerebral blood vessels altering blood flow. Ang II can cross the blood–brain barrier and contribute to reduced glutamate reuptake altering sympathetic output. Salt loading in rodents increases astrocyte release of ATP contributing to high blood pressure. Microglia can increase sympathetic output indirectly by release of pro-inflammatory cytokines or directly by decreasing expression of PDGFB thereby altering K^+^ channel biology in neurons

Although general neuroinflammation is important in CVD pathology [7], we lack a comprehensive understanding of the cell-specific changes to neuroimmune niches during CVD. Here, we will review the major functions of these neuroimmune niches and their role in CVD (Fig. 1). However, we will not include a discussion on neural control of the peripheral immune system, since it has been extensively reviewed elsewhere [8–10].

## Immune Surveillance of the Central Nervous System

Although microglia are the most abundant brain-resident immune cell, both microglia and astrocytes serve as the main immunocompetent cells within the CNS parenchyma. Both cell types are capable of cytokine release [11, 12], phagocytosing cell debris and toxic aggregates [13–15], and expression of major histocompatibility complex class II (MHCII) for antigen presentation [16, 17]. Indeed, these glial cells leverage their extensive arborizing processes to surveil their immediate neuronal and vascular environments. Specifically, microglia surveil the brain by extending and retracting their processes [18] while astrocytes form a syncytium with neighboring astrocytes to monitor neurotransmission [19] and vascular tone [20]. Reciprocal communication between microglia and astrocytes is necessary for the clearance of toxic protein aggregates. For example, microglia and astrocyte co-cultures clear amyloid-beta (Aβ) and α-synuclein more effectively than either monoculture alone [21]. Tunneling nanotubes mediate a direct and physical communication between microglia and astrocytes to facilitate microglial clearance of protein aggregates from astrocytes [21]. This reciprocal communication for the clearance of brain waste products is necessary, as the accumulation of toxic protein aggregates in the brain is linked to CVD and dementia [22].

Adjacent to the parenchyma, the brain is endowed with a specialized subset of myeloid cells residing in the choroid plexus, leptomeninges, and perivascular spaces. Collectively, these three populations of macrophages are known as brain border associated macrophages (BAMs). Although BAMs arise from the same yolk-sac-derived erythro-myeloid progenitor cells as microglia, the two cell types diverge by embryonic day 12.5 [23].

BAMs in the leptomeninges and perivascular spaces establish a stable and self-renewing population with minimal exchange from blood-derived cells, whereas macrophages in the choroid plexus undergo continuous exchange with peripheral hematopoietic stem cells (HSC) [24]. A recent study found that in the absence of disease, BAMs play major roles in regulating arterial vasomotion, extracellular matrix remodeling, and CSF flow [25••]. While much remains to be explored regarding the homeostatic actions of BAMs, evidence supports a role for BAMs in CVD including cerebral amyloid angiopathy [26•] and hypertension-induced neurovascular dysfunction [27]. Moreover, BAMs were recently identified as key contributors to Aβ-immunotherapy-induced microhemorrhages [28•].

The dura is the outermost and thickest layer of the meninges and serves as a major site of lymphatic drainage for the CSF [29]. Dural immune cells, particularly sinus-associated antigen-presenting cells, are uniquely positioned to detect brain-derived antigens found in the CSF flowing through the dural sinuses [30••]. Functional dural lymphatic vessels [31] drain these brain-derived antigens and immune cells to the deep cervical lymph nodes [32]. The dural neuroimmune niche comprises innate and adaptive immune cells that are vastly heterogenous both in their cellular make up and spatial distribution. Though largely predominated by macrophages, a wide variety of immune cells can be found throughout the dura, including neutrophils, B-cells, T-cells, natural killer cells, and dendritic cells [30••]. These immune cells are spatially localized to different sub-compartments depending on their function. For example, dural macrophages are closely associated with dural blood vessels [33]; T cells are predominantly found surrounding the dural sinuses [30••]; and pro-, pre-, immature, and mature B cells are found adjacent to and within dural blood and lymphatic vessels [34•, 35]. Fenestration of dural vessels allows for a large portion of dural immune cells to be trafficked from the periphery [36], including IgA-producing plasma cells educated in the gut and trafficked to the meninges to prevent CNS infection from blood pathogens [37].

Studies over the past 5 years have demonstrated the existence of direct channels between the dura and skull bone marrow [38••, 39••, 40••]. The discovery of dura-to-skull bone marrow channels suggests that brain-derived antigens in the CSF can influence HSC expansion and immune cell function in the skull bone marrow [40••, 41•]. Particularly, the skull bone marrow has been found to supply the dura with myeloid cells, including monocytes, neutrophils, dendritic cells, and macrophages [38••] as well as B cells and B cell progenitors [35]. These recent findings are paving the way for future discoveries, and much remains to be determined. For example, the cues that regulate the functional communication between the dura and skull bone marrow, and particularly the effect of disease on this communication, require further investigation.

## Brain Immune Niches and CVD

### Brain Parenchyma: Astrocytes and Microglia

A growing body of literature supports that astrocytes contribute to blood pressure elevation, a major risk factor for developing several types of CVD [42]. To this point, astrocytes in the hypothalamus can detect changes in diet and in turn cause major alterations to their neighboring vasculature. For example, in response to a high-fat/high-sugar diet, astrocytes secrete vascular endothelial growth factor (VEGF), causing an increase in hypothalamic angiogenesis and a thickening of the endothelial cell basement membrane prior to the development of hypertension [43]. In the paraventricular nucleus of the hypothalamus (PVN), reactive astrocyte-specific release of adenosine triphosphate (ATP) contributes to osmotic sensing and signaling after salt loading [44], which may increase blood pressure by promoting purinergic sympathoexcitation [45]. PVN astrocytes also respond to angiotensin II (Ang II), a hormone involved in human hypertension, by decreasing glutamate reuptake subsequently increasing the basal firing rate of PVN neurons and sympathetic output [46]. Inhibition of the Ang II receptor type 1 (AT1R) mitigates the effects on astrocyte reactivity [47].

Astrocytes are also crucial to the proposed mechanisms of brain waste clearance. In the glymphatic system model, glia provide the unidirectional waste clearance via convective fluid transport that is mediated by aquaporin-4, an astrocyte-specific water channel [48–50]. Alternatively, in the intramural peri-arterial drainage (IPAD) model, spontaneous smooth muscle contractions drive the clearance of soluble waste along the basement membrane of capillaries and arteries [51]. Computational modeling demonstrated that increased astrocyte coverage of an arteriole could also increase IPAD [52]. Despite the debate as to the relative importance and contribution of these two mechanisms, both are relevant to CVD, as astrocyte dysfunction leads to accumulation of waste products promoting neurotoxicity and neurodegeneration [53]. For example, impaired CSF flow and reduced solute in young spontaneously hypertensive rats (SHR) coincides with a decrease in parenchymal astrocyte area in the cortex [54].

Microglia also participate in the pathogenesis of hypertension. PVN microglial activation and pro-inflammatory cytokine release are associated with blood pressure elevation in various models of hypertension [55, 56], as well as myocardial infarction [57]. The pathology of both conditions is attenuated by treatment with minocycline, a non-specific inhibitor of microglial activation [55, 58], or by complete depletion of microglia [59]. Factors regulating neuroinflammation were also found to attenuate the cardiovascular pathology in both heart failure and hypertension, such as hypothalamic overexpression of the anti-inflammatory cytokine IL-10 [55], infusion of TGF-β into the cerebral ventricles [60], or the specific blockade of pro-inflammatory signaling [61]. More recently, the communication between microglia and neurons was found to be a key mediator of blood pressure elevation [62••]. In this study, microglia-derived PDGF-B acts via neuronal PDGFRα to regulate the basal sympathetic tone of PVN pre-sympathetic neurons [62••].

Single-cell RNA sequencing from multiple independent researchers has consistently shown that microglia do not express AT1R [63–68]. Paradoxically, although microglia lack AT1R, they become activated in response to Ang II stimulation [59]. This may be an indirect pro-inflammatory effect of Ang II on nearby neurons. Alternatively, microglia may begin to express the AT1R following a primary insult [69, 70•], which interacts with the toll-like receptor 4 (TLR4) receptor to promote neuroinflammation [71, 72]. However, other members of the renin-angiotensin system (RAS) may also drive microglial responses; for example, prorenin stimulation induces a pro-inflammatory response [56], whereas stimulation with angiotensin-(1–7) can elicit anti-inflammatory effects [73].

In short, astrocytes are capable brain resident sensors and actuators in response to peripheral signals that contribute to hypertension and metabolic disease. Microglial activation states and microglia-derived cytokines are clearly important for CVD progression. However, much remains to be determined regarding the astrocyte-to-microglia bidirectional communication in CVD.

### Parenchymal Borders: Border Associated Macrophages

BAMs reside at the brain borders and serve to protect and support the interface between brain and periphery [74]. BAMs are continuously exposed to CSF due to their localization and likely monitor and regulate the CSF milieu. Indeed, BAMs rapidly phagocytose molecules such as dextran and ovalbumin delivered into the brain or the cerebral ventricles [75]. They also control CSF flow by regulating arterial vasomotion [25••], which is relevant for conditions like cerebral amyloid angiopathy, a progressive accumulation of amyloid in the leptomeninges and superficial cerebral vessels [76]. The hypothesis that amyloid accumulation in cerebral blood vessels is the result of reduced brain clearance [77] is supported by the fact that BAM depletion increases vascular amyloid deposits in mice that express a mutated form of the amyloid precursor protein [78]. Moreover, ablation of BAMs reduces CSF flow in 2-month-old 5xFAD mice and worsens amyloid accumulation, indicating that BAMs are necessary for amyloid clearance [25••]. Similarly, BAM depletion exacerbates tau pathology in the PS19 transgenic mouse model of tauopathy [79]. Yet, there seems to be a limit as to how much toxic waste can be cleared by BAMs. For instance, exposure to excess amyloid leads to toxic production of reactive oxygen species (ROS) by BAMs, thus impairing neurovascular function [80].

BAMs are also critical in mediating the amyloid-related imaging abnormalities (ARIA) that follow anti-Aβ immunotherapy [28•]. BAMs are activated by exposure to immune complexes and contributed to disruption of the blood–brain barrier (BBB) through modulation of the basement membrane [28•]. Notably, BAMs also regulate BBB disruption in hypertension [81] and stroke [82]. In various models of hypertension, BAMs and particularly AT1R activation in BAMs contribute to BBB disruption [82] as well as impairment of neurovascular and cognitive function [27, 83]. In stroke, BAM-derived VEGF contributes to BBB leakage and worsened neurological function [83].

Hypothalamic BAMs can also respond to peripheral signals. Following myocardial infarction, they modulate sympathetic activation in response to increased circulating levels of TNF and IL-1β [84]. It has also been suggested that BAMs can modulate sympathetic activation in hypertension [85]. In obesity, hypothalamic BAMs were also found to upregulate inflammatory cytokines IL-1β, IL-6, and TNFα, to exacerbate astrogliosis and promote breakdown of the BBB [86]. BAMs also play an important role in regulating the hypothalamic–pituitary–adrenal axis in systemic inflammation [87–89], as well as in chronic stress [90].

Recently, efforts have been placed on understanding the heterogeneity of BAMs subpopulations [91], such as those expressing Lyve1 [92•, 93••] and MHCII [25••]. This is particularly interesting, because it explores the possibility that these subpopulations function in different ways and may even serve different roles in health and disease. In addition to unraveling the distinct roles of BAM populations, it is equally important to decode the crosstalk between BAMs and neighboring cells, such as endothelial cells and glia.

### Meninges and Skull Bone Marrow: The Next Frontier

A major risk factor for cardiovascular disease and several neurodegenerative diseases is aging, which affects dural immunity and meningeal lymphatic function. Specifically, aging impairs meningeal lymphatic drainage leading to the accumulation of macromolecules in the CSF that may contribute to cognitive decline [94, 95]. Changes in lymphatic endothelial cells, including increased expression of vascular cell adhesion molecule 1 (VCAM1) [30••] and altered expression of gene sets involved in immune and inflammatory responses [94, 96•, 97], are associated with the impaired meningeal lymphatic drainage in aging.

The link between the immune system and impaired meningeal lymphatic drainage was recently uncovered by a study showing that T cell accumulation in the aged meninges altered the lymphatic endothelial cells’ response to interferon-γ (IFNγ), thus impairing meningeal lymphatic function [97]. Interestingly, in aged mice, T cells not only surround the dural sinuses but are also found throughout the dura [30••], suggesting a shift in the signaling mechanisms required for homing and retention of these cells. Indeed, dural T cells in aged mice have reduced expression of CCR7, an important receptor necessary for mediating the lymphatic drainage of these cells, and deletion of *Ccr7* leads to neurovascular and cognitive impairment [98•]. Furthermore, aging increases meningeal FOXP3^+^ Tregs, which contribute to decreased amyloid clearance and increase cognitive deficits in 5xFAD mice [98•]. Beyond T cells, aging is also associated with an accumulation of B cells [35, 99] and a reduction in B cell progenitors in the dura [100]. Thus, meningeal immunity is critical for maintaining meningeal lymphatic function and proper brain waste clearance.

Cerebrovascular events, such as ischemic stroke, increase γδT17 cells in the leptomeninges [101]. We recently found that γδT17 cells producing IL-17 in the dura mediate the neurovascular and cognitive impairment in salt-sensitive hypertensive mice [102]. Through mechanisms yet to be discovered, the arachnoid barrier underwent significant tight junction remodeling allowing IL-17 to enter the CSF [102]. Thus, as several cytokines can affect and modulate neuronal function [2], our finding that the arachnoid barrier is disrupted in hypertension has wide ranging implications, as it represents an entry path for peripheral molecules to reach the central nervous system and affect brain function in CVD.

Although the immune system in the dura and skull bone marrow are linked, they remain largely understudied in cardiovascular disease and to date, their communication has only been characterized in stroke models. Most notably, Herisson et al. demonstrated that skull bone marrow-derived neutrophils were more likely than tibia bone marrow-derived neutrophils to migrate to the site of injury after transient middle cerebral artery occlusion (tMCAO) [39••]. Considering the evidence for a skull-specific response to brain injury and functional dural immune changes due to blood pressure elevation, future research should explore the dynamic communication between the skull bone marrow and the dural immunity in the context of hypertension.

Little is known about the changes in lymphatic drainage during CVD. In stroke models, photothrombolysis but not tMCAO increases meningeal lymphangiogenesis, an effect that is modulated by VEGFR3 [103]. Lymphatic hypoplasia in Vegfr3^−/−^ mice does not affect the outcomes following photothrombolysis, yet it exacerbates stroke severity after tMCAO [103]. On the other hand, promoting lymphatic drainage after intracerebral hemorrhage improves behavioral performance and reduces the volume of the brain hematoma [104]. Although increased lymphatic drainage after stroke may have a protective effect, with or without lymphangiogenesis, the long-term effects of increased lymphangiogenesis on immune cell populations in the dura must still be determined.

## Conclusions

Here, we reviewed the growing body of literature demonstrating a functional relationship between the brain and immune system during health and in CVD. Although work detailing the interplay between the brain and immune cells is robust, there are still many knowledge gaps that remain to be addressed.

In addition to CVD, hypertension is a major risk factor for dementia and cognitive decline. In an Ang II model of hypertension, BAMs were a major source of ROS production resulting in reduced neurovascular coupling and ultimately cognitive impairment [27]. Recently, we demonstrated in a DOCA-salt model of hypertension that dural T-cell-derived IL-17 generates ROS production in BAMs via IL-17RA, which impairs neurovascular coupling and also leads to cognitive impairment [102]. Of note, in both studies, depletion of BAMs [27] or IL-17 T-cells [102] reversed cognitive impairment. These findings and others [26•, 98•] lay the groundwork for future studies to focus on the mechanisms that underlie the detrimental effects on neuronal function that result from brain border immune surveillance of peripheral to ultimately contribute to cognitive impairment and dementia.

Considering the newfound relationship between the dura and skull bone marrow, future research should investigate the hypertension-induced changes to skull bone marrow-derived immune cell populations and cytokine profiles. As mentioned above, dura to skull bone marrow communication has only been investigated in stroke models and remains poorly understood in CVD. Given the important contribution of CVD to cognitive impairment and dementia, this neuroimmune niche cannot remain unexplored. We recently found that γδT17 cells in the dura mediate the neurovascular and cognitive impairment in DOCA-salt hypertension [102], and IL-17 produced in the dura gained entry into the CSF through a disruption of the arachnoid barrier [102]. This discovery identifies a new entry path for peripheral molecules, such as Ang II, into the CNS.

CVD may also affect the relationship between microglia and astrocytes contributing to cognitive impairment. Here, we discussed the individual roles of astrocytes and microglia in blood pressure elevation. However, the two cell types are inexorably linked as they rarely function without the other and their relationship is often described as a double-edged sword. In one hypothesis, persistent microglial activation in response to neuronal damage causes the release of inflammatory cytokines that leads to neurotoxic astrocyte reactivity [104]. Further research is needed to determine how the bidirectional relationship between astrocytes and microglia exacerbates CVD and contributes to end-organ damage to the brain in chronic conditions such as hypertension. As a first step, existing single-cell RNA sequencing datasets should be leveraged to explore this communication using inference analysis such as CellChat [105].

The neuroimmune niches discussed in this review provide a new and intriguing focus for both preclinical and clinical researchers. Importantly, we must consider the function of these neuroimmune niches in regulating brain homeostatic function beyond immunity. For example, a single-nucleus RNA sequencing study revealed that choroid plexus epithelial express high levels of components from the renin angiotensin system [106••]. Considering that brain expression of renin is low [107], this recent discovery of renin-expressing cells in the choroid plexus [106••] could support the local production of Ang II in the brain. Filling the known and yet-to-be-identified knowledge gaps will be critical for mitigating the deleterious effects of CVD on a global scale.

## Data Availability

No datasets were generated or analysed during the current study.
